# The progress in quantitative evaluation of callus during distraction osteogenesis

**DOI:** 10.1186/s12891-022-05458-8

**Published:** 2022-05-24

**Authors:** Qi Liu, Ze Liu, Hongbin Guo, Jieyu Liang, Yi Zhang

**Affiliations:** 1 Department of Orthopaedics, Xiangya Hospital, Central South University, Hunan Province, Changsha, 410008 China; 2 National Clinical Research Center for Geriatric Disorders, Xiangya Hospital, Central South University, Changsha, Hunan China

**Keywords:** Distraction osteogenesis, Quantitative evaluation, Callus, Imaging, Biomechanical evaluation, Biochemical markers

## Abstract

The manual monitoring of callus with digital radiography (X-ray) is the primary bone healing evaluation, assessing the number of bridged callus formations. However, this method is subjective and nonquantitative. Recently, several quantitative monitoring methods, which could assess the recovery of the structure and biomechanical properties of the callus at different stages and the process of bone healing, have been extensively investigated. These methods could reflect the bone mineral content (BMC), bone mineral density (BMD), stiffness, callus and bone metabolism at the site of bone lengthening. In this review, we comprehensively summarized the latest techniques for evaluating bone healing during distraction osteogenesis (DO): 1) digital radiography; 2) dual-energy X-ray scanning; 3) ultrasound; 4) quantitative computed tomography; 5) biomechanical evaluation; and 6) biochemical markers. This evidence will provide novel and significant information for evaluating bone healing during DO in the future.

## Background

In the 1850s, Dr. Ilizarov put forward the theory of "tension-stress" and the biomechanical principle of distraction osteogenesis (DO) [1]. The regeneration signal system of living tissue is activated with a continuous, stable and slow distraction force, which stimulates the division of cells and tissue regeneration. Under a physiological stress force, bone and its attached muscles, fascia, blood vessels and nerves will also grow synchronously. This technique could repair and reconstruct severely damaged limb tissues through self-repair and regeneration and treat complex orthopedic disorders (challenging to treat by traditional orthopedic techniques) [2]. DO is an effective and decent treatment for significant bone defects, limb shortening, bone nonunion, severe limb deformities, and severe neurovascular skin injuries [3].

However, the complications of DO should not be ignored, such as nonunion or delayed healing of bone, distraction injury of soft tissue, issues of force lines in lengthening areas and dislocation of adjacent joints [4, 5]. Indeed, it is not easy to complete limb lengthening and achieve desired clinical results. Therefore, it is imperative to monitor the process of bone lengthening, especially for the callus in the lengthening area. Monitoring bone healing can help clinicians better identify how well or poorly the new bone is healing to take early action (e.g., slowing down lengthening) to promote bone healing. One of the disadvantages of using an external fixator is that it is less comfortable and affects the quality of life. Prolonged use of an external fixator increases the chance of nail tract infection and osteoporosis. Monitoring the maturation of new bone can guide clinicians to remove the external fixator as early as possible, which is essential to prevent degenerative bone fractures and deformation and provide clinicians with a reference standard for removing the external fixator. It would be interesting to explore more accurate, convenient and inexpensive quantitative monitoring methods to facilitate proper decision-making by clinicians and benefit patients; thus, monitoring the bone healing process would be meaningful.

**Fig. 1:**
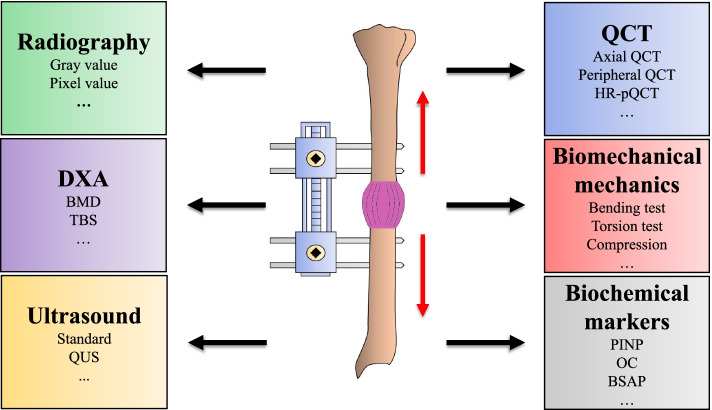
Different Methods for monitoring the process of bone healing

Currently, a variety of evaluation methods have been employed to monitor bone lengthening, including X-ray image analysis, dual-energy X-ray absorptiometry (DXA) quantitative computed tomography (QCT), ultrasound, biomechanical evaluation and biochemical markers (Fig. 1) [6, 7]. An X-ray examination is the most traditional and standard monitoring method [8]. It was always judged by the number, thickness of bone texture and BMD. However, it is easily affected by X-ray projection dose and image quality, and the sensitivity is poor. Therefore, the direct use of X-ray cannot accurately evaluate and predict the maturity and mechanical strength in distraction areas. In addition, the repeatability between the readers is also poor [9, 10]. However, the alteration of BMD or morphological structure of new bone tissue will affect the repair efficacy of DO and determine whether the new bone tissue can meet the functional requirements. Therefore, the method of evaluating new bone tissue is fundamental. Each evaluation method has its advantages and disadvantages, and it is necessary to choose an appropriate evaluation method in experimental or clinical work. Babatunde et al. reviewed the appropriate evaluation methods in 2010 [7, 11]. However, they mainly focus on imaging evaluation, and biomechanical evaluation and biochemical markers were not considered. Most importantly, since many novel advances have been made in the field recently, it is essential to comprehensively summarize the evidence for bone healing evaluation. Therefore, this review will discuss the progress in quantitative evaluation of calluses during DO.

### Standard radiography (X-ray)

#### X-ray evaluation of new bone formation

The X-ray imaging technique is still an essential method for the clinical evaluation of bone healing. Bridging callus at least 2 mm thick shown in three of the four cortices on plain anteroposterior and lateral radiographs, is one of the radiological standards for external fixator removal in limb lengthening [12, 13]. In addition, the clinical standards to remove the external fixator are listed as follows: 1) After loosening the upper and lower nuts of the ring fitting fixed by the threaded bar 0.5 cm, the patient could bear weight without abnormal sensations. 2) The fixed time is generally consistent with the average extension index (the total fixation time of the external fixator is the average time of soft callus consolidation calculated from the date of lengthening. Each 1 cm is fixed for 1 month, called the average extension index). However, these measurements are a subjective processes with high intra- and interobserver variability [9, 10].

#### Relationship between measurement parameters and new bone

Some researchers have tried to study quantitative methods for evaluating bone healing by X-ray imaging techniques. Scholars have mainly studied the related parameters of plain radiographs, such as the pixel value ratio (PVR) and gray value (GV). The pixel value evaluates BMD in pixels, which can be used to assess the healing of regenerated bone (compare the density of regenerated bone with that of adjacent bone). As the density of the regenerated bone increases with the healing, its pixel value is close to that of the adjacent normal bone. GV is another indicator used to evaluate bone healing. A grayscale image is a data matrix. Its value represents a specific range of brightness values, where brightness 0 means black and brightness 255 refers to white. The part of the grayscale image with intense luminance represents the object with high density or thickness; the part with weak light transmission represents the object with low density or thin thickness [14].

#### Related research progress

Song et al. [15] retrospectively analyzed 40 tibial segments of 23 patients with different limb lengthening indications. The Ilizarov method was used for bone lengthening after osteotomy, and digital X-ray and DXA scans were used to monitor distraction and fixation. Pearson correlation analysis was used to compare the correlation between PVR and the BMDR. The results showed a positive correlation between BMDR and PVR, indicating that PVR measured by X-ray could effectively evaluate callus hardness (especially in the case of callus maturation and suspected callus hardness). Zhao et al. [16] reviewed 17 patients (34 lengthenings) who underwent bilateral tibial intramedullary nail lengthening, and analyzed the PVR of the lengthening area on plain radiographs. They suggested that PVR on plain radiographs can be used as an objective parameter for callus measurement and guide the timing of external fixator removal. They also suggested that when the PVR of the two cortices was 1, patients could partially use crutches to carry weight; when the PVR of the three cortices was 1, patients could carry weight without crutches at all. However, they did not give how much cortical minimum PVR could safely remove the external fixator. Bafor et al. [17] investigated the role of PVR in determining the time of full weight-bearing in patients undergoing intramedullary limb lengthening, and they found no adverse effect when subjects began full weight-bearing (when the cortical PVR of 3/4 was at least 0.93). Vulcano et al. [18] also explored the PVR assessment of bone healing and found that the PVR was not affected by sex, age or lengthening. A PVR value of 0.90 can be considered bone healing. Zak et al. [19] proposed that PVR combined with subjective evaluation parameters (including continuity, signal intensity and homogeneity of regenerated tissue) was proposed in the X-ray Evaluation System for Distraction Osteogenesis (XESDO), which will be conducive to monitoring the bone healing in DO.

Vaccaro et al. [14] evaluated the accuracy of BMD by using the average gray value (MGV) of digital radiography in 11 cattle and 2 horses in vitro. The cattle specimens were imaged using conventional radiography, while horse specimens were imaged using digital radiography. Each sample was then scanned using the same DXA device. The BMD values obtained by each DXA were matched with the MGV from conventional X-ray or digital X-ray images. They found that the MGV analysis was accurate for BMD evaluation (the coefficients of variation were 0.10 and 0.09, respectively), and the correlation coefficients with DXA analysis were 0.910 and 0.937, respectively. Lucas et al. [20] indicated a correlation between the GV measured in digital X-ray images and the BMD measured by DXA. The BMD and GV of two femurs of 15 dogs were measured and quantitatively analyzed by DXA and routine X-ray. It was found that there was a good correlation between the GV measured in the selected X-ray setting condition and the average BMD measured by DXA (*r* = 0.61). It was possible to use digital radiography to measure fundamental alterations in BMD. However, the soft tissue was not considered.

#### New directions

The advantages of PVR in evaluating bone healing are that it is convenient and straightforward, it is easier to popularize, and it requires a lower radiation dose than DXA and CT. However, the above research has certain limitations.The evaluation method also needs to be compared with the effectiveness of the current standard for evaluating bone healing with plain radiographs, and the retrospective nature of the experiment, lack of a control group, and other potential factors affecting the calculation of PVR should also be considered in further studies.

On the other hand, evaluating BMD with GV is inexpensive and convenient, while its sensitivity is lower than DXA. More study is still needed to improve its value in evaluating callus healing. For example, the correlation between GV and callus biomechanics could be considered simultaneously. Overall, X-ray is a traditional method for monitoring bone healing, which is relatively simple, rapid and convenient (the first choice for clinicians). GV and PVR can provide an objective value for evaluating new bone. However, the potential information for callus formation and bone healing is limited [21]. It converts a three-dimensional object into a two-dimensional image and finally into a one-dimensional number, so it may miss a small cortical space, which may lead to fracture after the removal of the fixator. Therefore, X-ray and other imaging indicators may be a more accurate evaluation method in callus formation during DO.

### Dual-energy X-ray scanning

#### DXA evaluation of new bone formation

DXA uses X-ray radiation sources to emit two different radiation energies. By measuring their absorption through bones and soft tissues separately, the equal absorption part of bone tissue is calculated, thus, the influence of soft tissue is eliminated. Then, the BMC, and aBMD (area bone mineral density) of bones in all body parts can be measured and the changes in bone trabeculae can be observed [22], which is the most widely used and mature measurement technique of BMD. It is considered the gold standard for measuring BMD and diagnosing and treating osteoporosis [23]. BMD measurement can provide the basis for the Fracture Risk Assessment Tool (FRAX), a computer evaluation software established to evaluate fracture risk based on the measured BMD combined with fracture risk factors [24]. DXA can guide clinical work, helping doctors monitor bone mineralization and predict fracture risk. Clinically, Eyres et al. [11, 25] first described the use of DXA to quantify and monitor regenerative bone formation in leg lengthening and detected regenerative bone within 2 weeks after traction, in contrast to 6 weeks on plain radiographs. They suggested that DXA could monitor the number and rate of new bone growth. Therefore, DXA can be used to evaluate and monitor new bone formation during callus lengthening [11, 25], and it is an effective and reliable method for quantifying BMC of regenerated bone [26].

#### Relationship between measurement parameters and new bone

DXA measurements (including BMD, BMC, and vBMD) are well represented in terms of the strength of new bone, and many scholars hope to quantitatively monitor bone healing in patients with osteotomy lengthening with measurements obtained by dual-energy X-ray absorptiometry. Reiter et al. [27] studied 20 patients with a unilateral fixator's limb extension of the femur or tibia. They monitored the BMD of callus during and after extension,and they found that the maximum BMD occurred 4–6 weeks after the beginning of distraction. Futhermore, from the curing period of the callus to the removal of the fixator, the BMD of the distraction space increased continuously. Their results provide a basis for monitoring bone healing using DXA. Saran et al. [28] proposed that DXA could be used as a prediction tool to remove external fixators. They performed BMD analysis on a DXA scan once a month in 26 patients with limb extension. The external fixator was removed once the BMD was stable below 10%, and the plain radiography showed no apparent defects.Using a DXA scan to monitor bone healing during the extended consolidation phase, fractures after external fixator removal were very low (3.6%) while maintaining an acceptable bone healing index without excessively increasing the fixation time. Song et al. [15] studied the bone mineral density ratio (BMDR). BMDR is the ratio of the BMD value of the regeneration area to the average BMD value of the proximal and distal normal bone. They retrospectively analyzed 23 patients with 40 tibial segments undergoing limb lengthening. They concluded that it is safe to remove the fixator when the BMD of the regenerated bone reaches 51.1% of the reference cortical BMD. Recent studies have shown that DXA scanning is helpful to evaluate bone healing after DO, but more research is needed to monitor the unified standard of bone healing.

#### Related research progress

The BMD value adequately reflects the amount of bone mineral content, but it does not necessarily represent the level of bone strength and stiffness. Therefore, some scholars have researched the correlation between DXA scan measurements and bone biomechanical properties after DO in recent years, but such research results are still controversial. Tselentakis et al. [29] studied the BMD measured by DXA and the bending stiffness of distraction bone segments. They observed 9 patients with DO. Six weeks after distraction, the patients underwent regular DXA scans to monitor BMD and measure the bending stiffness of the distraction segments. They found a highly significant correlation (R2 = 0.77, *P* < 0.001) between the bending stiffness of the callus and the square of the total mineral content at the minimum BMD. Therefore, they concluded that DXA scan could be effectively used to determine the bending stiffness of the callus, and is valuable in determining the removal time of the external fixator and the delay in bone healing. Monsell et al. [30] explored the relationship between DXA assessment of regenerative bone and structural mechanical properties in an animal model of DO. They found significant correlations between vBMD and the elastic modulus, yield stress, and failure stress of bone, while no correlations were seen between BMC, BMD, vBMR, and other mechanical parameters. They concluded that DXA was a promising tool for assessing regenerative bone formed by DO during limb lengthening that required further investigation. Floerkemeier et al. [31] found that stiffness measurement was a better quantitative index of bone bearing capacity than BMD and BMC measured by DXA.They did not find a significant correlation between BMD and bone strength.

#### New directions

In conclusion, DXA can quantitatively monitor the distraction process and can also be used to decide when to remove the fixator. However, DXA scanning costs are high, and there are limitations in measuring the mass distribution of cortical bone and bone trabeculae and evaluating bone geometry and microstructure. The measurement effect is poor in the presence of artifacts, so it is not routinely used [32, 15]. More exploration and evidence are needed to support the correlation between DXA measurements and bone strength. It is believed that DXA will hopefully be used in more fields to exert its value in the future.

### Ultrasound

#### Ultrasound evaluation of new bone formation

Ultrasound is a non-invasive, effective and inexpensive method for evaluating bone healing [33]. The principle is that wide-band ultrasonic attenuation signals are employed to evaluate BMD alteration. When ultrasound passes through body tissue, attenuation occurs, and the amount of attenuation is related to the characteristics of the tissue. Using the speed of ultrasonic waves and amplitude attenuation passing through the bone can calculate the amount of BMC, bone structure and bone strength[34]. The evaluation of normal mature bone by ultrasound is limited by the inability of ultrasound to penetrate the bone cortex. However, new bone with incomplete remodeling and calcification can be well evaluated by high-resolution linear ultrasound [35]. Contrast-enhanced ultrasound (CEUS) can provide an early indication of neovascularization and provide a diagnostic basis for poor bone regeneration [36]. Ultrasound can also predict bone callus formation by observing changes in blood flow around the new bone [37], which can compensate for the poor early visualization of the callus on X-ray.

#### Relationship between measurement parameters and new bone

The beam penetration depth (BPD) measured by ultrasound monitors the filling of the bone extension section, with a lower BPD indicating better bone filling, which can provide helpful information for monitoring bone healing and when to remove the external fixation frame [39]. Researchers have found that various ultrasound parameters can be used to quantitatively evaluate new bone quality, which is very sensitive to the dynamics of bone growth [40–42]. Mesquita et al. [43] studied the ultrasonic propagation velocity and BMD before and after demineralization of sheep backbone cortical bone. They found a good correlation between ultrasonic velocity and BMD (*r* = 0.75956). Sorriento et al. [44] further investigated the quantitative measurement method of BMC by B-ultrasound. They prepared bone-like models with different concentrations of hydroxyapatite (HA) and calcium carbonate (CaCO3) evaluated by ultrasound, and utilized phase entropy calculated based on backscattering data, ultrasonic amplitude information, and absorption scattering phenomena to detect changes in BMC. Therefore, ultrasound seems possible to quantitatively evaluate BMD. However, the study did not use samples of natural tissues, including skin and blood vessels, which need to be further reinforced. Tang et al. [45] achieved good results in quantifying novel bone in a sheep tibial mass defect model using three morphological parameters of ultrasound (new-bone bulk, new-bone surface and new-bone contact).

#### Related research progress

Troulis et al. [46] estimated bone formation on X-ray and ultrasound using a semiquantitative bone fill score. They found that plain radiographs underestimated bone formation compared with ultrasound. Andrade et al. [39] conducted further research and found that the correlation between the ultrasound bone fill score and intraoperative bone fill score was higher than that of plain radiographs. Hence, they concluded that ultrasound is a technique that can accurately evaluate new callus formation.

Ultrasound can detect novel bone formation 4–6 weeks earlier than X-ray [47]. Neretin et al. [48] compared X-rays and ultrasound to evaluate the process of human metatarsal regeneration during distraction and fixation. Ultrasound presented an average of 9.0 ± 0.08 mm echo-positive regeneration space and the heterogeneous structure of regenerated bone on the 10th day of distraction. Slight echo-positive inclusions correspond to new bone formation, whereas X-ray could not reflect apparent regeneration. Notably, the formation of new callus in the early stage of distraction often predicts the trend of DO. Therefore, ultrasonic monitoring can provide early information on bone lengthening. Clinical doctors can accelerate or slow down the speed of distraction to acquire a decent clinical outcome (to prevent the occurrence of bone nonunion or delayed healing). Since sufficient blood vessels are one of the pillars of bone regeneration, local and systematic analysis of microperfusion of new bone provides a new and promising diagnostic method [49]. CEUS can show the microperfusion of local tissue at the capillary level. Through the study of patients with tibial nonunion, Haubruck et al. [36] found a relationship between the expression of different angiogenic cytokines in serum and the change in local microperfusion. They believe that the combined detection of CEUS and cytokine expression analysis (CEA) is a promising new tool for the early prediction of tibial nonunion. Augat et al. [37] used color Doppler ultrasound to continuously monitor the blood flow at the fracture site in fracture patients. They found that the callus was well-formed with abundant blood flow signals around the callus; however, if there was no blood flow signal around the callus, the callus did not form well. He et al. [50] used ultrasound to monitor patients after tibial transfer. They found that the size of the hematoma was linearly and negatively correlated with the bone healing time at the end of the osteotomy (*r* =—0.819, *P* < 0.01). They believed that ultrasound could detect the changing trend of hematoma, blood flow signal and callus around the osteotomy end earlier to provide timely and effective feedback. Young et al. [51] used ultrasound to monitor 12 patients treated with DO, of which 2 cases found liquid cysts in the traction area during traction, which would affect the formation of callus and delay the mineralization time of callus. Ultrasound monitoring was used to detect and extract light yellow liquid in time. Therefore, ultrasound monitoring of DO can significantly reduce the risk of complications such as poor osteogenesis and secondary fracture. Ultrasound has been widely popular for its low price, portability,lack of ionizing radiation and ease of operation [52].

#### New directions

Ultrasound monitoring of the bone lengthening process has its advantages, especially in the hematoma organization period in the early stage of distraction lengthening. Ultrasound can identifyphenomena that X-ray cannot detect. Simultaneously, ultrasound can monitor the new callus earlier, and evaluate the number of calli, which enables medical workers to adjust the speed of traction lengthening earlier according to the monitoring results (to prevent complications such as delayed healing and nonunion). Other complications, including vascular and nerve stretch injury and liquid cysts in the elongated area, can also be detected by ultrasound. Meanwhile, ultrasound is noninvasive, quick, convenient, inexpensive and does not require radiation. However, ultrasound has some limitations in monitoring bone mineralization in the late stage of distraction, which makes it challenging to guide the removal of the external fixator. In addition, the performance of the limb force line is not intuitive enough to measure the end gap of osteotomy in the latter stage. Although it is difficult to determine the generally applicable normal value of the quantitative ultrasound index and the diagnostic criteria, ultrasound has a broad application prospects in monitoring bone healing with the improvement of technology and the accumulation of clinical experience.

### QCT

#### QCT evaluation of new bone formation

QCT is a method to measure the human body's BMD, bone shape, and body composition by adding a QCT special phantom and analysis software based on a CT machine. This method has high accuracy and small error directly related to histological specimens. It is an excellent method to measure the alteration of long BMD. The principle is based on different tissues' different absorption of ionizing radiation. Doctors can compare attenuation measurements to standard reference values ​​using a standard computed tomography scanner ​​to calculate information such as BMC and BMD [53]. QCT can use three-dimensional imaging to evaluate calluses. It can also perform finite element analysis to predict the strength of calluses, which helps clinicians to fully understand the structure and growth of callus. With the development of CT technology, researchers have applied QCT in musculoskeletal research.

#### Relationship between measurement parameters and new bone

QCT can overcome the shortcomings of DXA, which cannot measure cortical bone and trabecular bone separately, and can measure BMC by whole-body thin-section tomography. Modern QCT can measure axial bone BMD and peripheral bone (pQCT), which is the only method for measuring actual BMD in three dimensions [32]. BMD in QCT is measured as volumetric bone mineral density [vBMD] in grams per cubic centimeter, whereas DXA measures area bone mineral density (aBMD) in grams per square centimeter [53]. QCT also measures the strength-strain index (SSI), which is calculated from the geometric and material properties of the bone and is a prediction of the mechanical strength of the bone [54]. Harp et al. [55] demonstrated a strong correlation between the apparent density of canine tibial specimens and QCT BMD measurements. The authors derived an equation that accurately predicted the hardness of tubular bone using measurements collected by QCT. Engelke et al. [56] reported the correlation between BMD and BMC measured by QCT and DXA through spiral CT scanning of the forearm. They proposed that the whole-body spiral CT scanner can conduct a high-precision density evaluation of the distal radius. The bone mineral density measured by QCT and DXA is positively correlated with BMC (*r* = 0.55 ~ 0.80).

#### Related research progress

In recent years, several scholars have proposed new methods for assessing bone healing by QCT. Swennen et al. [57] improved the evaluation of bone healing by QCT and proposed a new method. They distracted the skulls of 16 sheep and evaluated the regenerated bone by three-dimensional quantitative computed tomography(3D-QCT) and conventional QCT. They found that 3D-QCT correlates with conventional QCT, and the measurement of BMD based on 3D-QCT is effective and reliable for evaluating bone healing during DO. QCT can measure the BMD of trabecular and cortical bone (close to the quantitative measurement of volume BMD in the real sense) [32]. Bone healing participates in recovering both original tissue structure and biomechanical function. QCT has been utilized in the evaluation of biomechanical properties and bone strength [58–61]. Kokoroghiannis et al. [54] studied the correlation between pQCT bone strength index and mechanical tests in DO. They found that a significant positive correlation between the bone strength index and the maximum load during bone destruction, and the correlation coefficient was 0.846 (*P* < 0.001). This shows that the bone strength index measured by pQCT can be used as a sensitive index for the complete solidification of regenerated bone. Bone strength can be determined by mechanically testing the ultimate load of bone fracture. However, this method can only be employed in vitro. On the other hand, by using the density-modulus equation derived from experiments, finite element analysis could predict bone strength in vivo [62]. Dailey et al. [63] scanned a group of patients with tibial shaft fracture by three-dimensional CT reconstruction. They performed virtual mechanical tests by finite element method to evaluate the torsional stiffness of fractured limbs, indicating that virtual mechanical testing with low-dose CT scanning could provide quantitative and objective evaluation of callus structure. To compare the accuracy of mechanical properties predicted by density-modulus equations of different finite element analyses, Vijayakumar et al. [64] measured the mechanical properties of bone by an indentation test on the tibia of 5 cadavers. The predicted bone strength varied with different anatomical areas, whereas the Goulet density-modulus equation was the best method.

#### New direction

Overall, QCT is a decent and reliable method for detecting bone healing. It reflects volumetric BMD, can measure the growth of regenerated bone relatively accurately, and can monitor changes in bone mass early. It also provides high-resolution three-dimensional imaging of the callus and a quantitative analysis of the area to help clinicians objectively assess whether the bone has healed enough to remove the external fixator. The measured value of QCT can also reflect the change in bone biomechanical strength. Nevertheless, its high cost and large radiation dose should also be considered, and the range of its application is not wide enough at present. More studies are still needed to address the issues above. With the development of technology, QCT assessment of bone healing will provide more valuable information, such as monitoring bone healing with high-resolution pQCT and predicting the strength of new bone by finite element analysis.

### Biomechanical evaluation

#### Biomechanical evaluation of new bone formation

Measuring the changes in the mechanical properties of bone is the most direct method to evaluate the process of bone healing. Bone biomechanics is based on the theory of engineering mechanics, which evaluates bone quality by the mechanical properties of bone tissue under external action and the biological effect of bone after stress [65]. The bending, torsion, tension, and compression tests are commonly used to evaluate the mechanical properties of distraction osteogenic new bone tissue [66, 67]. Mechanical parameters of bone such as bending stiffness and torsional stiffness are helpful to understand the consolidation of new bone. They are essential parameters reflecting the strength and elasticity of bone and directly reflect the quality and quantity of new bone in bone healing. Understanding the mechanical properties of new bone can help prevent new bone fractures and guide clinicians on when to remove external fixators. In the clinic, the biomechanical properties of new bone can be measured using relevant instruments for stiffness measurement [68, 69]. Dwyer et al. [69] studied 30 lengthened calves and found that when the tibia reached 15 Nm/ or the femur reached 20 Nm/, there was no further fracture. This shows that the mechanical detection of newborn bone can provide valuable information for clinical workers.

#### Relationship between measurement parameters and new bone

The mechanical strength and stiffness of callus as a healing index have been generally accepted. Strength is the maximum bearing capacity of an object at the moment before it is damaged by the loading force, which can only be measured in the laboratory. Stiffness is a structural mechanical property of matter expressed by the ratio of the loading force on the object to the deformation displacement produced by the structure. Floerkemeier et al. [31] studied sheep bone specimens after DO. They found that the biomechanical parameters of regenerated bone obtained from torsion, compression, bending tests were better quantitative indicators of bone-bearing capacity than BMD and BMC measured by DXA.

The bending is used to determine the bending stiffness and the ultimate strength (the maximum bearing capacity of a body at the moment before its failure by the loading force) of the new bone tissue by measuring the load /deflection, which can objectively and indirectly reflect the maturity of the new bone tissue. This evaluation should be compared with normal bone under the same conditions to avoid excessive bias, which is commonly employed to evaluate the biomechanics of calluses in the laboratory. Chotel et al. [70] evaluated the bending test of 11 children who underwent leg lengthening surgery. They obtained the reference value of bone bending stiffness in limb lengthening healing through statistical analysis. The torsion test involves clamping the two ends of the tested sample in the torsion test machine, driving the machine to apply torque to the sample, recording the maximum load of damage after the sample is destroyed, and simultaneously recording the load and bone deformation in the instrument. Thus, bone tissue's ultimate strength and torsional stiffness can be determined [71, 72]. This assessment could provide a better biomechanical index of bone. Floerkemeier et al. [73] studied 26 sheep with tibial distraction osteogenesis. Their studies showed that torsion, bending, and compression stiffness measurements were suitable for predicting callus healing load-bearing capacity. However, the measurement of torsion stiffness was slightly better than that of compression stiffness and bending stiffness. The tension and compression experiment involved placing the sample in the experimental machine, and stretching the sample at a constant speed or applying compressive stress to the sample until the sample was destroyed. The system will automatically output the maximum load, stress, strain, and elastic modulus data [74], and it can be used as an index for the biomechanical evaluation of bone.

#### Related research progress

Recently, some novel techniques have been reported to evaluate the biomechanical properties of bone, such as micron indentation and nanoindentation technology. These technologies can be utilized to evaluate the hardness of regenerated bone [75]. By placing a tapered diamond probe hardness tester on a smooth bone surface with a preset time and load, the hardness value changes with the dynamics of the indentation depth. After the load is removed, the diagonal indentation or width is recorded. The hardness of the bone can be calculated according to the geometric calculation of the diamond probe [76, 77]. Researchers are trying to develop a noninvasive biomechanical detection method for its clinical application [78, 79]. Recently, in vivo monitoring technology has emerged, mainly focusing on the changes in mechanical parameters with time during the mineralization of new tissues [80–83]. Liu and Aarnes et al. [84, 85] proposed that the axial load sharing ratio can be used to indirectly evaluate the hardness of calluses as the basis for safe removal of external fixators. Mora Macias et al. [82] also proposed a new distractor that can detect the axial stiffness of calluses.

#### New directions

Measuring the mechanical properties of bone is the most direct method to evaluate the process of bone healing, and accurate data for bone biomechanics can be obtained. It can also provide a basis for finite element analysis and quantitative evaluation of the effectiveness of callus healing. Combining bone mechanical property test data with imaging parameters will be conducive to assessing callus healing. However, the damage to the bone in the process of measurement is a severe limitation. Currently, these methods are only used in laboratory research. The application of in vivo mechanical monitoring technology expands the understanding of DO. It provides information for developing anumerical model for the mechanical characterization of callus as a future clinical tool [86–89]. With the deepening of the research, it is reasonable to believe that the biomechanical detection of bone is promising as a practical and straightforward method to evaluate the process of bone healing during DO in the future.

### Biochemical markers

#### Biochemical marker evaluation of new bone formation

Theoretically speaking, the alteration of bone metabolism may lead to subsequent morphological changes. In other words, the changes in biochemical marker should be earlier than those in BMD monitoring. Therefore, biochemical markers are a potential novel evaluation method for bone healing, and could be a decent supplement to imaging examinations in clinical work.

#### Relationship between measurement parameters and new bone

At present, several kinds of biochemical markers of bone transformation (BTMs) have been identified [90], such as osteocalcin (OC), bone specific alkaline phosphatase (BSAP), N-terminal procollagen peptide (PINP), and C-terminal procollagen peptide (PICP) [91, 92], and they can reflect the biological activities of osteoblasts and osteoclasts in vivo.

#### Related research progress

Windhagen et al. [93] found in the sheep DO model that OC began to increase in the distraction phase and peaked in the curing phase. Fink et al. [94, 95] further studied the relationship between BTM and radiographic density during DO. They found that the average correlation coefficient between OC and radiographic density was 0.68 ± 0.11, and they also observed that PICP levels increased rapidly after surgery and further during consolidation. Therefore, they believe that measuring serum OC and PICP levels can obtain valuable information about bone formation during DO. Leung et al. [96] studied the goat DO model and found a strong correlation between plasma BSAP activity and newborn bone's radiological morphology and biomechanical properties. This shows that we can use BSAP to monitor the change process of callus formation. Kumar et al. [92] prospectively studied 168 patients with closed tibial fractures treated with interlocking intramedullary nails. The treatment process was divided into six periods to determine BTMs (BSAP, PINP, OC, etc.) and clinical and imaging evaluation. They found that bone formation markers (BSAP, OC, PINP) were significantly lower in patients with delayed union. Therefore, continuous monitoring of BTMs can be served as an auxiliary means for imaging examination (predictingdelayed healing).

#### New directions

Bone metabolic markers have been reported to monitor bone healing, and some of them have a high degree of theoretical feasibility. The biochemical bone markers detected in different diseases, osteogenic distraction sites, and treatment methods vary, complicating the issues. Some indicators can be monitored by serum or urine samples, and different methods limit the comparability between different studies. Combining different biochemical markers in bone healing during DO is also a good form of evaluation. Overall, the clinical data for biochemical markers are somewhat limited. Further well-designed experimental and clinical studies are still needed.

## Conclusion

There are several ways to evaluate bone healing during DO quantitatively. In this study, we comprehensively reviewed the methods of X-ray, DXA, ultrasound, QCT, biomechanical detection and biochemical markers in the quantitative evaluation of bone healing. Various methods are irreplaceable and complementary to each other in monitoring the whole process of bone lengthening, and each of them has its advantages and disadvantages (Table 1). Considering multiple monitoring methods simultaneously may be a better solution, which might be the future direction for clinical bone lengthening (e.g., combining imaging evaluation and biochemical markers). With progress in the field, more optimized evaluation method for bone healing during DO will be considered in the future.

**Table 1:** The advantage and disadvantage of each assessment for bone healing

Method	Advantage	Disadvantage
Radiography	Simple, fast and convenient; The most common assessment for bone union	Limited sensitivity for early callus formation
DXA	Golden standard for BMD; Indirect assessment for bony microstructural	Expensive; Limited for clinical application; Limited for limb assessment
Ultrasound	Cheap, portable, ionizing radiation free and early monitoring	Relatively limited sensitivity; Limited access to the force line of limb; Unaccessible for later osteotomy end space
QCT	High accuracy and small error; Directly related to histological specimens	Expensive; High radiation dose; Limited for clinical application
Biomechanical evaluation	Directly assessed the bone quality	Usually utilized in lab work
Biochemical markers	Theoretical basis for delayed bone union	Limited for clinical application

## Data Availability

NA.
